# Histopathology aiding diagnosis of viscerocutaneous loxoscelism in a nonendemic region

**DOI:** 10.1016/j.jdcr.2023.12.014

**Published:** 2024-01-05

**Authors:** Smrithi Mani, Charles Katzman, Vincent Liu

**Affiliations:** a Carver College of Medicine, University of Iowa Hospitals and Clinics, Iowa City, Iowa; b Department of Dermatology, University of Iowa Hospitals and Clinics, Iowa City, Iowa; c Departments of Dermatology and Pathology, University of Iowa Hospitals and Clinics, Iowa City, Iowa

**Keywords:** brown recluse, clinicopathologic correlation, spider bite, viscerocutaneous loxoscelism

## Introduction

Viscerocutaneous loxoscelism is a rare syndrome caused by brown recluse spider envenomation.^1^ In addition to the cutaneous manifestation of a necrotic wound, it can present with systemic symptoms, including acute hemolytic anemia, renal failure, disseminated intravascular coagulopathy, and even death.^1^, ^2^, ^3^ The *Loxosceles reclusa* is responsible for most cases of loxoscelism in the United States.^2^ Diagnosis and management of viscerocutaneous loxoscelism can pose challenges because of the often undetected initial bite and limited studies on treatment.^3^ We report a delayed diagnosis of fulminant viscerocutaneous loxoscelism in a nonendemic region supported by histopathology.

## Case report

A previously healthy 19-year-old Black man was transferred to our intensive care unit for an acute drop in hemoglobin. In the week before admission, the patient presented to his local emergency department for fever, malaise, and vomiting. A lesion behind his left knee was noted, initially considered unrelated and benign. He was diagnosed with a viral illness and sent home. The day before transfer, he returned to the emergency department for worsening symptoms and a syncopal episode. The lesion behind his knee had become tender and swollen. Computed tomography of the left lower extremity was consistent with cellulitis. Given clinical concern for sepsis, he received clindamycin before his hemoglobin dropped to 3.4 g/dL. He was stabilized with 2 units of packed red blood cells and transferred.

Admission laboratory results showed hemolytic anemia. Etiology was unknown at the time. The patient received intravenous immunoglobulin and dexamethasone while the hematology and infectious disease services conducted their investigations. During initial hospitalization, he experienced significant respiratory distress secondary to pulmonary edema, gross hematuria, and laboratory studies showed transaminitis, leukocytosis, and significantly elevated inflammatory markers (Table I).

**Table I:** Longitudinal laboratory values, important events, and workup during hospitalization

Laboratory tests∗	Hospital day							
	1	2	3	4	5	6	7	8
Blood count								
White blood cell count (K/mm^3^)	33.37	41.7	39.1	39.1, 43.2	32.7	17.1	12.6	8.7
Hemoglobin (g/dL)	3.4, 4.1, 6.5	6.0, 7.3, 7.0	7.0	6.9, 9.1	8.4	8.9	8.4	8.8
Platelets (K/mm^3^)	165	-	-	69	58	53	59	-
Hemolysis laboratory results								
Total bilirubin (mg/dL)	14.5	4.4	1.9	-	-	1.3	1.2	1.1
PT/INR (s/IU)	13/1.2	-	-	-	-	-	-	-
Lactate dehydrogenase (μ/L)	1601	-	-	-	-	767	599	-
# Reticulocyte count (K/mm^3^)	48.0	-	-	-	-	390.9	-	-
Haptoglobin (mg/dL)	<10	-	-	-	-	<10	13	31
Kidney/liver function								
AST (μ/L)	45, 142	144	45	-	-	29	22	25
ALT (μ/L)	30, 107	107	68	-	-	57	49	47
BUN (mg/dL)	24	21	20	21	20	18	16	15
Creatinine (mg/dL)	1.08	0.86	0.92	0.91	0.90	0.90	0.86	0.83
**Important events**		Diffuse desquamating rash; pulmonary edema		Seizure-like episode				
**Treatment**	IVIG (1 g/kg, 2 doses), 2 units of PRBCs, solumedrol, dexamethasone (40 mg, 4 doses), levetiracetam (started on day 4), several antibiotic courses (clindamycin, doxycycline, ceftriaxone, metronidazole, vancomycin)							
**Workup for alternateetiologies (allinsignificant)**	*Anaplasma phagocytophilum* (Ab and PCR); *Ehrlichia chaffeensis*, *Ehrlichia ewingii*, *Ehrlichia muris*-like (Ab and PCR); Lyme disease (Ab); MRSA; blood culture, *Staphylococcus aureus* PCR, Legionella antigen, acid-fast bacilli culture, fungal culture, Giemsa stain, Malaria panel, Histoplasma antigen, Blastomyces antigen, Syphilis Ab, HIV (Antigen/Ab and PCR), Chlamydia PCR, Gonorrhea PCR, Hepatitis A/B/C Ab, EBV Ab, CMV PCR, Cardiolipin Ab, ANCA, ANA, Antistreptolysin, DNase B Ab, ADAMTS-13, Copper, Folate, B12							

*Ab*, Antibody; *ALT*, alanine transaminase; *AST*, aspartate transaminase; *BUN*, blood urea nitrogen; *EBV,* Epstein-Barr virus*; IVIG*, intravenous immunoglobulin; *PCR*, polymerase chain reaction; *PRBCs*, packed red blood cells, *PT/INR*, prothrombin time/international normalized ratio.

∗ Normal ranges: white blood cell (3.5-10.5), hemoglobin (13.2-17.7), platelets (150-400), total bilirubin (<1.2), PT (9-12), INR (<4.0), lactate dehydrogenase (135-225), # reticulocyte (12.0-130.0), haptoglobin (30-200), AST (0-40), ALT (0-41), BUN (10-20), creatinine (0.67-1.17).

**Fig 1:**
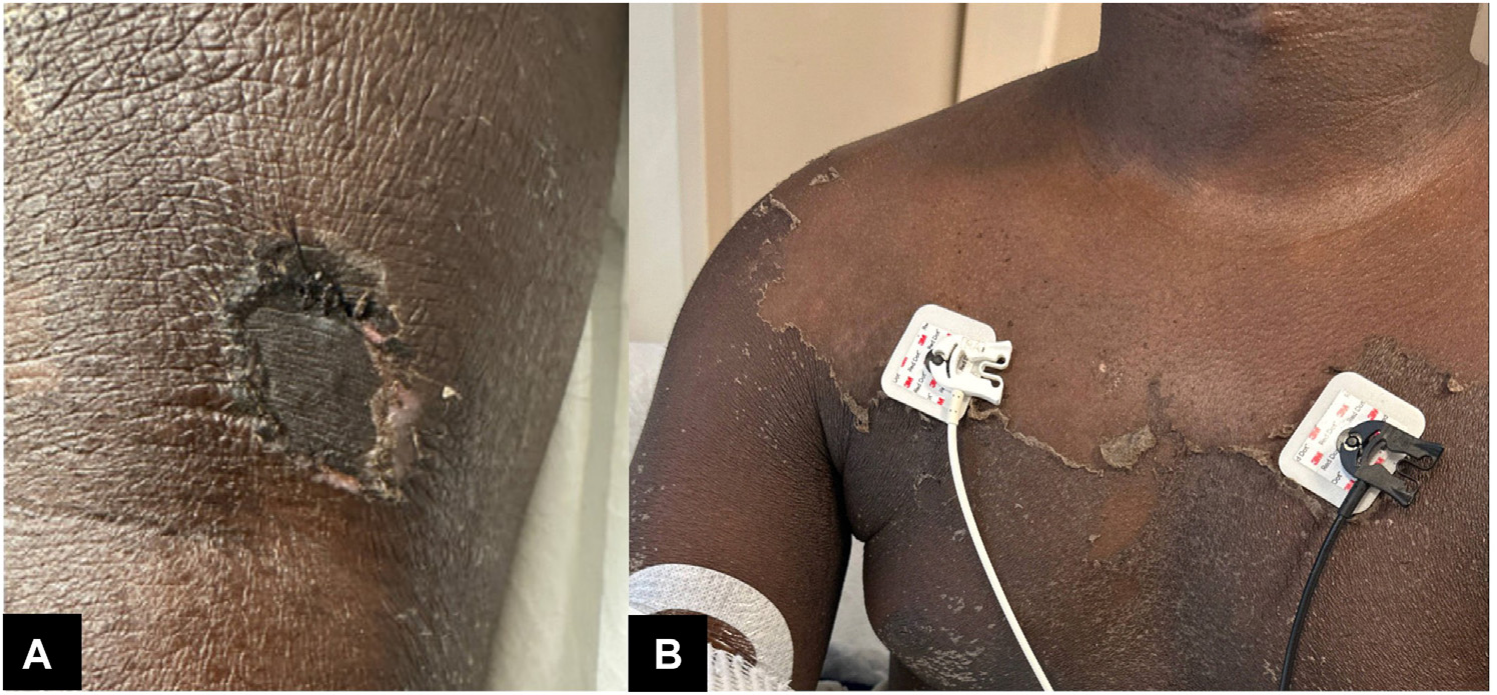
**A,** Necrotic eschar in the left popliteal fossa. **B,** Diffuse desquamation of the chest.

On day 2 of hospitalization, a widespread desquamating rash developed on his trunk, face, and extremities. Dermatology was consulted for the rash and the lesion in the left popliteal fossa (Fig 1). On interview, the patient had noted a “scratch” in the left popliteal area 1 week before hospitalization, which was itchy but not painful. He did not recall a definite spider bite but mentioned noticing spiders in their home in Iowa and sleeping on a floor mattress. The lesion had developed into a depressed, ulceronecrotic, atrophic, hyperpigmented plaque with scalloped borders, eschar formation, and surrounding induration.

**Fig 2:**
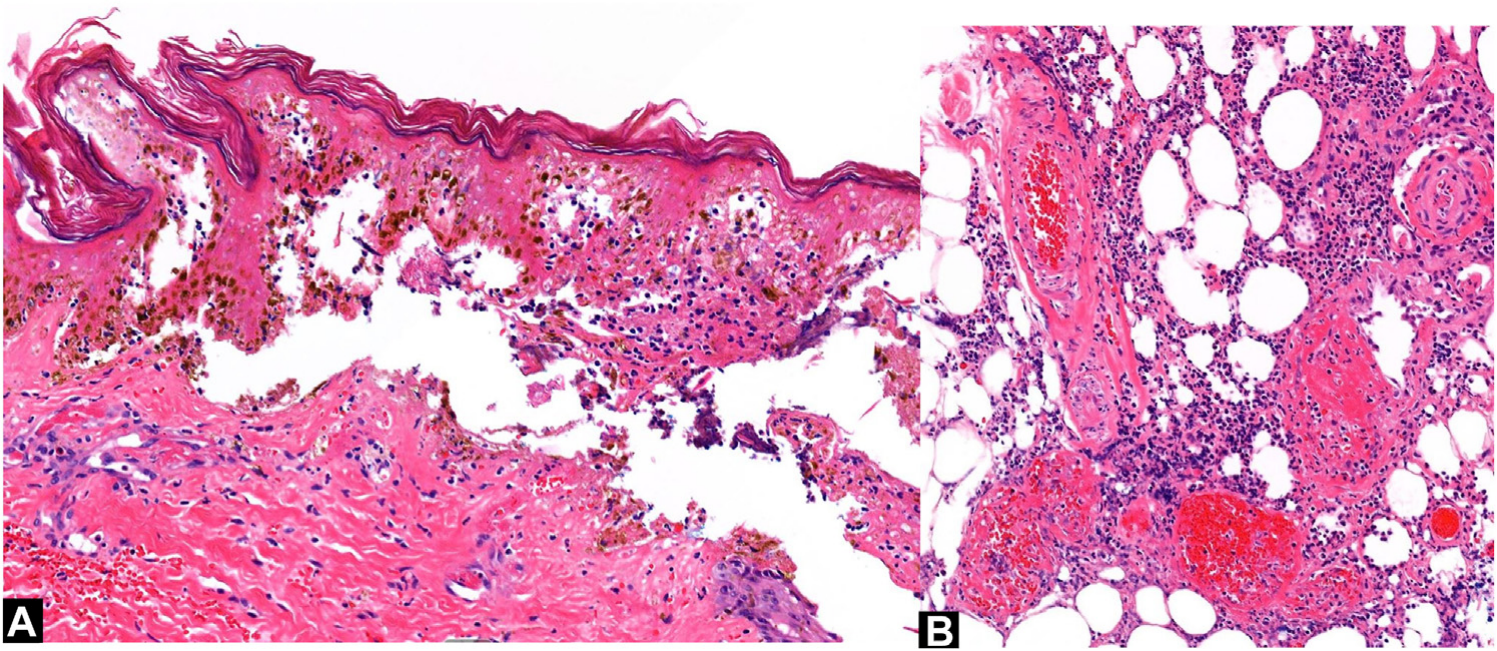
Histopathology of skin biopsy of left popliteal eschar. **A,** Epidermal necrosis (hematoxylin-eosin stain; original magnification: ×400). **B,** Neutrophilic vasculitis of pannicular blood vessels (hematoxylin-eosin stain; original magnification: ×200).

After our interview and physical examination, we obtained a single punch biopsy from the superomedial edge of the eschar. Histopathologic examination of the biopsy showed epidermal necrosis, superficial and deep neutrophilic vasculitis with vessel wall destruction, fibrin deposition, and karyorrhectic debris surrounding the dermal vessels (Fig 2). Special histochemical stains for acid fast, Gram, and periodic acid–Schiff, as well as immunohistochemical stains for spirochetes, herpes simplex virus, and varicella-zoster virus were negative for microorganisms. Histopathologic changes were deemed compatible with venomous arthropod assault.

The constellation of his unexplained symptoms with the necrotic appearing eschar, in conjunction with his laboratory and histopathologic data, was consistent with a diagnosis of viscerocutaneous loxoscelism. His diffuse skin sloughing was also attributed to loxoscelism.

Extensive workup for alternate etiologies was unrevealing (Table I). Direct Coombs testing was weakly positive. As his anemia improved, dexamethasone was discontinued. He experienced another syncopal episode, but all neurologic and cardiac workup was unremarkable. Ultimately, on hospital day 8, he was discharged.

## Discussion

We present this case of a previously healthy 19-year-old Black man with dermatologic findings of a necrotic eschar and widespread desquamating rash and systemic findings of acute hemolytic anemia, transaminitis, and pulmonary edema. Our patient’s florid systemic reaction, constellation of cutaneous findings, striking laboratory data, clinicopathologic correlation, and unusual geographic exposure history expands our appreciation of the presentation spectrum of viscerocutaneous loxoscelism. Although our patient could not provide physical evidence of a brown recluse spider from his home, the pattern and timing of his clinical symptoms coupled with the negative workup for alternate etiologies favor the diagnosis of viscerocutaneous loxoscelism over other infectious, autoimmune, or neoplastic causes (Table I).

Definitive diagnosis of loxoscelism requires confirmation of a bite and patient presentation of the offending spider for identification^1^; however, these criteria are not met in approximately 90% of suspected spider bite cases.^2^ Although there is an enzyme-linked immunosorbent assay available to detect *Loxosceles* venom, it is not commercially available and is primarily for research.^4^ Therefore, the primary suspicion for loxoscelism usually derives from report of a sensation of a possible bite. In about 40% of loxoscelism cases, including ours, there is no definite reported bite.^1^ An initial bite may go unnoticed, as it is fairly painless and commonly occurs at night.^1^, ^2^, ^3^

Given the elusive nature of diagnostic confirmation of loxoscelism, our case uniquely features the value of histopathologic data in corroborating a loxoscelism diagnosis, which is more accessible than enzyme-linked immunosorbent assay testing and provides a more conclusive assessment beyond clinical suspicion alone. Our histologic findings favor brown recluse envenomation over alternative arthropod assault because of the neutrophilic vasculitis, consistent with the study of 41 skin histologic specimens from rabbits injected with brown recluse spider venom.^5^ Histologic sampling from the rabbit wound site consistently showed epidermal and dermal necrosis, surrounding mixed inflammatory infiltrate with neutrophils, and small vessel vasculitis, aligning with our findings.^5^

*L reclusa*’s native range historically encompasses central and southeastern states.^2^ Awareness of this range aids in early detection for patients within endemic regions and helps to avoid potential misdiagnoses in nonendemic areas.^2^ However, our patient whose exposure in a nonendemic area which primarily lies outside the native region, highlights the importance of caution in reliance of geographic exposure, and underscores the critical utility of familiarity with the clinical features of loxoscelism for physicians.^2^ The diagnosis of viscerocutaneous loxoscelism in the state of Iowa is exceptionally rare, with the last published case being in 1980.^6^

Our patient had a particularly severe case of systemic loxoscelism, with his hemoglobin reaching a nadir of 3.4. The primary team was uncertain about the etiology of his anemia, possibly because of the infrequency of loxoscelism in nonendemic regions or challenges in identifying the cutaneous manifestations in individuals with darker skin tones. However, the dermatology team successfully diagnosed the condition by observing the eschar and widespread skin desquamation. This distinct cutaneous pattern associated with loxoscelism has previously been described in other patients with skin of color.^7^^,^^8^ Acute generalized exanthematous pustulosis has been reported as another diffuse rash associated with loxoscelism.^9^

Diagnosis of viscerocutaneous of loxoscelism can be useful to avoid unnecessary treatment for the many mimics including infection, necrotizing vasculitides, and neoplastic disease.^9^ Treatment of loxoscelism is controversial given cutaneous and systemic symptoms should resolve on their own.^1^^,^^3^^,^^9^ Various approaches have been trialed, but their effectiveness is inconclusive.^1^, ^2^, ^3^^,^^9^ Supportive care is the general recommendation in North America.^3^

Autoimmune hemolytic anemia is the characteristic feature of viscerocutaneous loxoscelism.^1^, ^2^, ^3^ Other systemic manifestations are relatively heterogenous but include neurologic symptoms, transaminitis, and pulmonary edema our patient experienced (Table II).^10^ *Loxosceles* spider venom contains an array of protein factors that contribute to the cutaneous and systemic manifestations of loxoscelism including hyaluronidase, alkaline phosphatase, esterase, ATPase, and sphingomyelinase D.^3^ Sphingomyelinase D, the predominant toxic enzyme, hydrolyzes sphingomyelin found in cell membranes, contributing to both dermonecrosis and hemolysis of red blood cells.^3^

**Table II:** Cases of viscerocutaneous loxoscelism in the United States∗

Study	Patient	Race	State	Diagnosis	Treatment†	Abx	Dermatologic features‡	Clinical features§
Edwards et al^6^	61 M	C	IA	Clinical	Steroids	Y	Gangrene of eyelids	Severe laryngeal edema with upper airway obstruction, seizures
Cain et al^11^	25 M	-	KS	Clinical	Steroids, plasma exchange	Y	-	ICU transfer
Abraham et al^12^	17 F	-	KS	Clinical	Steroids, plasma exchange	Y	-	ICU transfer
DiPaola et al^13^	19 M	C	KY	Clinical	Steroids	Y	-	Rhabdomyolysis, acute kidney injury, transaminitis
Langner et al^10^	16 M	B	MO	ELISA for *Loxosceles reclusa* venom	Steroids, IVIG, plasmapheresis	N	-	Myocarditis, pulmonary edema, cardiogenic shock, hypotension requiring ICU care, DIC, transaminitis
Mueller et al^9^	25 F	SA	MO	Clinical	Steroids, IVIG	Y	AGEP with extensive desquamation	Transaminitis, ICU care for severity of anemia
Neverman et al^14^	16 M	B	MO	Clinical	-	Y	-	Transaminitis
Harry et al^15^	49 M	C	MO	Clinical	Plasmapheresis	Y	-	Renal failure requiring hemodialysis, transaminitis
Calhoun et al^16^	30 M, 28 F	B	MO	Clinical	Steroids	Y	-	Transaminitis, acute kidney injury, spontaneous abortion
Schmid et al^7^	24 F	B	MO	Clinical	-	Y	Diffuse desquamating rash	Pleural effusion, pericardial effusion, cANCA/antiprotease-3 vasculitis
Schilli et al^17^	79 M	C	MO	ELISA for *L reclusa* venom	Steroids	Y	-	Obtundation, syncope, myocardial infarction
Anwar et al^18^	20 M	B	MO	Clinical	Steroids, IVIG	Y	AGEP	Hypoxic respiratory failure, transaminitis, pancreatitis, cardiomyopathy, ICU transfer
Lane et al^19^	9 M	B	MO	Clinical (spider available)	-	N	AGEP	Transaminitis, ICU transfer, high output heart failure, respiratory distress
Stoecker et al^20^	16 M	-	MO	ELISA for *L reclusa* venom	Steroids	N	-	Transaminitis
Kodali et al^21^	34, 31, 21, 21, 25, 22	-	MO	Clinical	-	N	“Diffuse rash”	Hepatosplenomegaly, syncope, necrotizing fasciitis, hypotensive shock requiring ICU care
Lane et al^22^	19 F, 9 F	B	MO	Clinical	Steroids	Y	-	-
Said et al^23^	6 M	B	MO	Clinical	Plasma exchange	N	-	DIC, renal failure, hypotensive shock requiring ICU care
Zimmerman et al^24^	12 F, 9 F	B	NC	Clinical (spider available)	Steroids	Y	Scarlatiniform rash	Pulmonary edema, toxic shock syndrome
Dandoy et al^25^	10 M	B	TN	Clinical	Steroids	N	-	HLH, splenomegaly, transaminitis, renal failure requiring hemodialysis
Rosen et al^26^	3 M	C	TN	Clinical	-	N	-	Death
McDade et al^27^	17 F, 16 M, 13 M, 12 F, 13 F, 14 F	B	TN	Clinical	-	N	Desquamation surrounding necrotic wound	Severe hypotension requiring ICU care
Donepudi et al^8^	11 M	B	TN	Clinical	-	Y	Desquamating, depigmenting rash on face, neck, and trunk	-
Elbahlawan et al^28^	15 M, 15 F, 3 M, 12 M, 10 M, 11 M	B	TN	Clinical	Dapsone, steroids	Y	Diffuse desquamating rash	Pulmonary edema, renal failure requiring hemodialysis, severe hypotension, ICU transfer
Williams et al^29^	26 F, 17 F	B	TN	Clinical	Steroids	Y	Fine papular rash of the extremities, extensive skin sloughing extending from necrotic wound	Death, pulmonary edema, pleural effusion, pericardial effusion, hepatic congestion
Rogers et al^30^	11 M	C	TN	Clinical	-	Y	-	ICU transfer
Michaud et al^31^	6 M	C/K	TN	Clinical	-	N	Diffuse erythematous, pruritic macular rash with petechiae	-
Hogan et al^32^	11 M	B	TN	Clinical	Steroids	Y	Tiny papules from umbilicus spreading outward	
Robb et al^33^	49 F	-	TN	Clinical (spider available)	Steroids, dapsone	N	Sunburn-like, purpuric eruption	
Wilson et al^34^	24 F	C	TX	Clinical (spider available)	Dapsone, steroids, hyperbaric oxygen therapy	N	Fine macular rash over trunk and extremities	-
Goto et al^35^	7 M	-	TX	Clinical (spider available)	Steroids, dapsone	Y	-	Upper airway obstruction secondary to edema, ICU transfer

*Abx*, Antibiotics; *AGEP*, acute generalized exanthematous pustulosis; *B*, Black; *C*, Caucasian; *DIC,* disseminated intravascular coagulation; *ELISA*, enzyme-linked immunosorbent assay; *F*, female; *IA*, Iowa; *ICU*, intensive care unit; *IVIG*, intravenous immunoglobulin; *K*, Korean; *KS*, Kansas; *KY*, Kentucky; *M*, male; *MO*, Missouri; *N*, no; *TN*, Tennessee; *TX*, Texas; *Y*, yes.

∗ This table includes case reports and series from the United States based on search of “systemic loxoscelism” and “viscerocutaneous loxoscelism” on PubMed, EMBASE, and Cochrane databases. Studies that were retrospective reviews, abstracts only, or did not have full text were not included.

† Treatment does not include supportive measures such as fluid resuscitation or blood transfusion.

‡ Dermatologic features do not include necrotic wound itself.

§ Clinical features do not include hemolysis or nonspecific constitutional symptoms. Of note, 78% (29/37) of patients in case reports with data on race were Black.

Viscerocutaneous loxoscelism can be a difficult diagnosis, especially in a nonendemic region where there is no reported bite and symptoms resemble more common disease processes. We highlight specific cutaneous and histopathologic features to aid in this challenging diagnosis.

## Conflicts of interest

None disclosed.
